# Selective Adsorption of Gadolinium by Nitrogen-Doped Carboxymethylated Cellulose Nanocrystalline Carbon Aerogels Functionalized in the Ammonia–Urea System

**DOI:** 10.3390/molecules28247965

**Published:** 2023-12-06

**Authors:** Tongtong Xu, Xudong Zheng, Ang Li, Biao Ji

**Affiliations:** School of Environmental Science and Engineering, Changzhou University, Changzhou 213164, China; xttbtt@163.com (T.X.); la122240180@gmail.com (A.L.); jjjj13952044190@163.com (B.J.)

**Keywords:** carbon aerogel, rare-earth elements, adsorption, recovery, gadolinium

## Abstract

In this paper, an ammonia–urea system was developed to induce the shedding of carboxymethylcellulose carbon aerogels to form defects, and the specific surface area of the aerogels was significantly increased after carbonization, and the three-dimensional disordered pore structure of cellulose was preserved. The material showed the selective adsorption of gadolinium ions using the carboxylate active sites provided by carboxymethylation and the microporous or mesoporous structures formed after carbon burning. The successful synthesis of the material was demonstrated by relevant characterization, and the results of static adsorption experiments showed that the material was more consistent with the quasi second-order kinetic model at pH = 5.0. The maximum adsorption capacity was 99.65 mg g^−1^. The material showed a high adsorption capacity for gadolinium ions in the presence of competing ions and maintained 84.07% of the adsorption performance after five adsorption cycles. The simple use of urea ensured that the cellulose maintained its pore structure, and the specific surface area was greatly increased after carbonization, which provided a feasible direction for the industrial adsorption and recycling of rare-earth elements for reuse.

## 1. Introduction

In “Made in China 2025” [1], it is mentioned that the key manufacturing fields, such as high-end equipment manufacturing, new energy industries, new material industries and new energy automobile industries, are closely related to rare earths. Rare earths are known as the “mother of new materials” and directly affect the development speed and level of each field [2]. In order to ensure the security of rare-earth resources, the United States and the West are vigorously promoting the rare-earth supply chain and industrial “de-Chinaization” process, trying to reconstruct the complete rare-earth industry chain with the United States and the West as the main providers [3]. In order to alleviate this problem and effectively protect rare-earth resources, the government has introduced a number of export bans and readjusted the tax rates for rare-earth resources, which has led to high prices for rare earths [4]. Since the implementation of the dual carbon strategy, the demand for electric motors has become stronger and stronger, and most of the rare-earth elements are magnetic, which makes them an important research material for the development of electric motors [5]. Gadolinium, as a low-cost, rare-earth element replacing the high-priced neodymium element in NdFeB permanent magnets, has a wide range of uses in downstream sectors, such as contrast agents for magnetic resonance imaging, optical glass, phosphors, and NdFeB permanent magnets (replacing the high-priced neodymium element) [6]. Along with the continuous development and utilization of gadolinium in the world, waste from rare-earth gadolinium has been generated, which not only destroys the living environment, but also leads to the abandonment of some gadolinium elements [7]. This requires the recovery of gadolinium from the waste; the current method of gadolinium recovery is mainly based on precipitation, the ion exchange method, the flotation method, and other methods [8]. The content of gadolinium in the waste is generally relatively low and contains other interfering elements, making selective recovery difficult and costly [9]. In this study, we chose a low-cost [10], biomaterial that is widely available in nature to fabricate carbon aerogels for the selective adsorption of gadolinium elements [11].

Cellulose is nature’s most widely and cheaply available biomass material [12], and white stable cellulose nanocrystal suspensions (CNCs) can be obtained by the acid hydrolysis of cellulose [13,14]. The surfaces of CNCs are mainly hydroxyl groups, and these groups cannot fulfill the adsorption separation of gadolinium elements [15,16]. In order to increase the adsorption amount and adsorption efficiency, we introduced a large number of carboxymethyl groups on the surface of CNCs by carboxymethylation to make carboxymethyl cellulose nanocrystals (c-CNCs) [17].

In our previous studies, the specific surface area of the aerogels was relatively low. Therefore, in this experiment, we carbonized the aerogels in a tube furnace to increase the specific surface area. However, cellulose aerogels can easily destroy their own three-dimensional pore structure during carbonization [18]. We used an ammonia–urea system to protect the cellulose aerogel; ammonia plays an important role in the formation of carbonization exfoliation defects in cellulose aerogels [19]. Ammonia plays an important role in the formation of defects in cellulose aerogels by carbonization exfoliation, which ensures that the endopores can maintain the formation of individual fibers in a honeycomb shape during the carbonization pyrolysis process, forming cellulose aerogels with a highly specific surface area [20]. The aim of our study is to obtain a cost-effective and promising green adsorbent material for the recovery of Gd^3+^ from rare-earth wastewater. In this experiment, we first used medical absorbent cotton to make CNCs using the existing method and carboxymethylated them to obtain c-CNCs. The ammonia–urea system was obtained by mixing urea with ammonia using simple mechanical stirring; then, the ammonia and urea were mixed with c-CNCs and freeze-dried to obtain cellulose aerogel, followed by carbonization at 450 °C in a tube furnace to obtain nitrogen-doped cellulose carbon aerogels (c-CNCs@N) [21]. In our experiments, the ammonia–urea system was utilized to protect the cellulose carbon aerogels, which induced the formation of a honeycomb pore structure of the individual fibers during the carbonization process and greatly provided the adsorption effect and adsorption capacity. We employed a series of characterizations to evaluate the aerogels quantitatively and qualitatively [22,23]. The adsorption experiments systematically investigated the effect of different factors (pH, contact time, temperature, and initial concentration) on the adsorption of Gd^3+^.

## 2. Results and Discussion

### 2.1. Characterization Results

#### 2.1.1. SEM Analysis of Aerogel Materials

**Figure 1:**
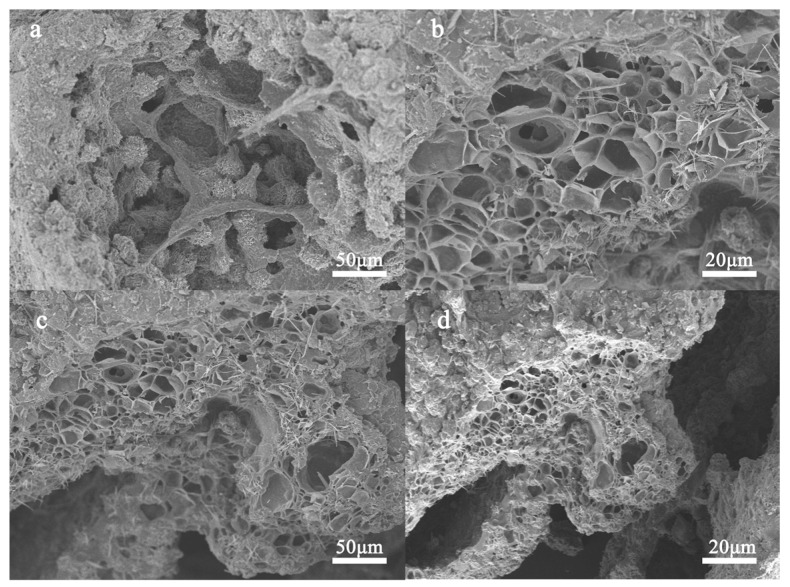
Scanning electron micrographs of (**a**,**b**) c-CNCs and (**c**,**d**) c-CNCs@N aerogels at different magnifications.

The surface morphology of the two aerogels was studied by SEM. As shown in Figure 1a,b, which shows the SEM photos of c-CNCs at different magnifications, it can be seen that there is a good pore structure, but the pore distribution is not uniform. And Figure 1c,d shows SEM pictures of c-CNCs@N at different magnifications, which demonstrate that the internal collapse and honeycomb pore morphology are ensured due to the ammonia–urea system. And the honeycomb pore morphology contributes to the ultra-lightweight property of the material, while increasing the specific surface area and enhancing the adsorption property of the material.

#### 2.1.2. FTIR Analysis of Aerogel Materials

**Figure 2:**
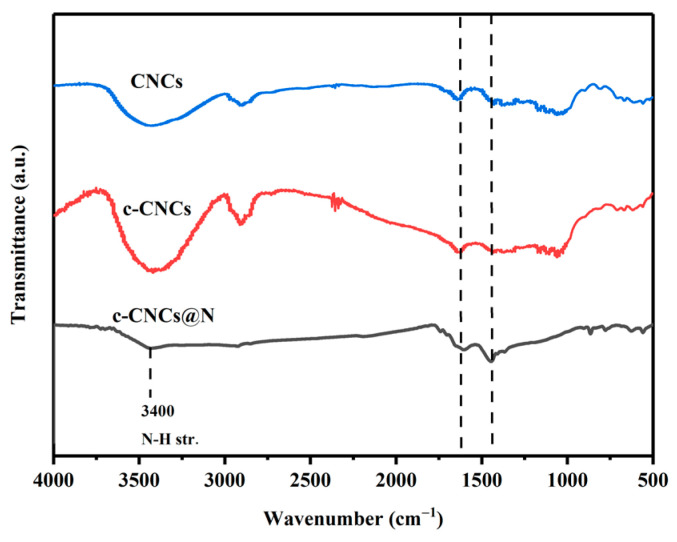
FTIR of CNCs, c-CNCs, and c-CNCs@N.

We conducted experimental tests in the range of 4000–500 cm^−1^ (FTIR) for the CNC, c-CNCs, and c-CNCs@N, respectively, and the results are shown in Figure 2. It can be seen from the figure that there are obvious peaks at 2902 and 1337 cm^−1^, which are caused by C-H stretching vibration and C-O-H bending, respectively, which are also characteristic peaks of cellulose. A characteristic peak of CH_2_COONa was found at 1610 cm^−1^, which indicates the successful synthesis of the carboxymethyl cellulose nanocrystals [24]. And for c-CNCs@N, the peak becomes smaller at 3650–3300 cm^−1^, which is due to the break of O-H during carbonization, and there is a sharp peak at 3400 cm^−1^, which is attributed to the vibration of N-H. In summary, the successful synthesis of c-CNCs@N was confirmed.

#### 2.1.3. N_2_ Adsorption–Desorption

**Figure 3:**
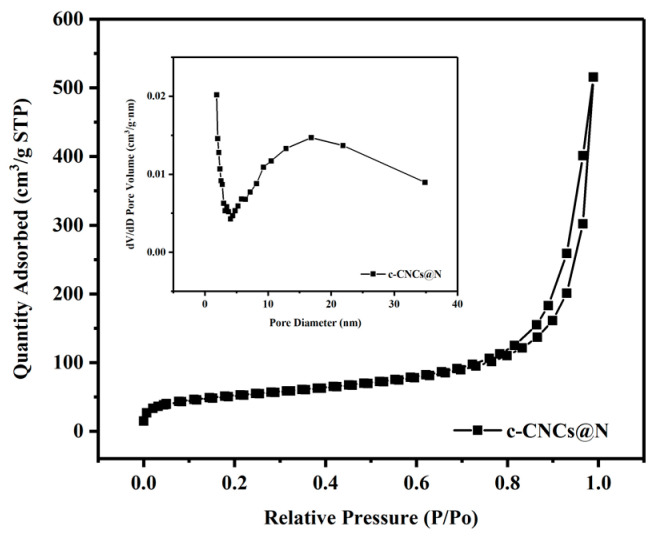
N_2_ adsorption–desorption isotherms of c-CNCs@N.

We conducted experiments on the aerogel materials by the N_2_ adsorption–desorption analysis method to investigate the pore size as well as the structure of the aerogels and to obtain the specific surface area of the aerogels, and the results are shown in Figure 3. It can be seen from the figure that the curve rises during the monolayer adsorption process when the relative pressure is between 0 and 0.8, while the adsorption and resolution curves become steep and hysteresis loops appear when the relative pressure is between 0.8 and 1.0. The specific surface area of the c-CNCs@N aerogel material is 176.98 m^2^ g^−1^, which indicates that the ammonia–urea system plays an important role in carbonation, protecting the pore channels and promoting the formation of collapses. The distribution of pore size is shown in the small panel in Figure 3. The pore size is mainly in the range of 0–40 nm, with a concentration around 5–20 nm, and the pores are micro-controlled or mesoporous, which is favorable for the adsorption of gadolinium ions.

#### 2.1.4. Zeta Potential Analysis of Aerogel Materials

**Figure 4:**
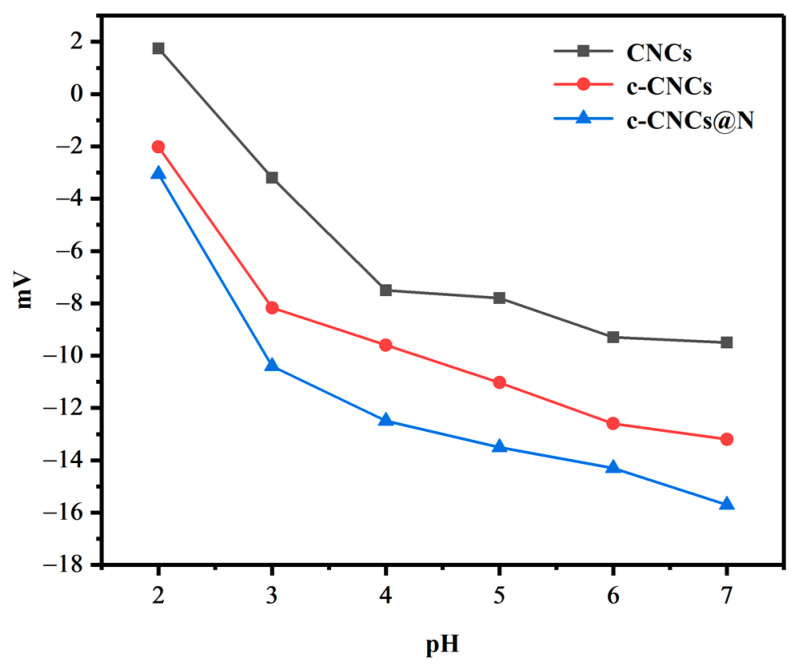
Zeta potentials of CNCs, c-CNCs, and c-CNCs@N.

The pH solution was adjusted using 0.1 M hydrochloric acid, and the surface charge distribution of the aerogel material was tested at different pHs because rare-earth ions form precipitates under alkaline conditions, so we controlled the pH value between 2.0 and 7.0 for the three aerogel materials, and the data results are shown in Figure 4, from which it can be seen that different pH values have different effects on the surface charge of the materials; CNCs has the smallest charge at pH 7.0. The overall surface charge of CNCs@N is low compared to that of the c-CNCs, which is probably due to calcination and nitrogen doping. In conclusion, a pH of 7.0 is the best theoretical condition for the adsorption of cations on this composite.

### 2.2. Adsorption Experimental Analysis

#### 2.2.1. pH Experiments

**Figure 5:**
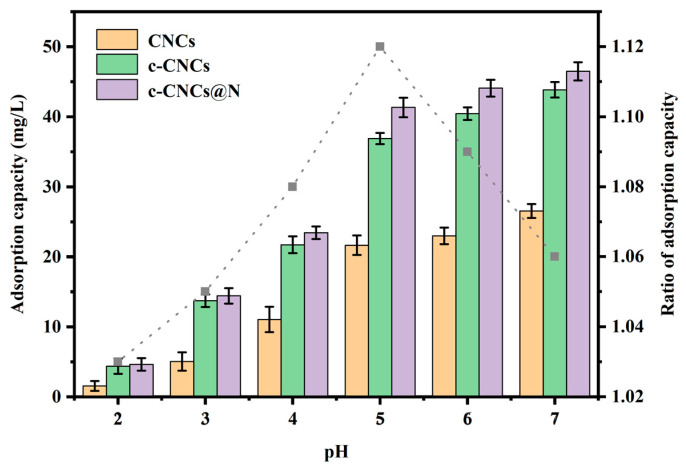
Optimum pH values of CNCs, c-CNCs, and c-CNCs@N aerogel adsorbing Gd^3+^.

In order to investigate the optimal pH of aerogel materials for Gd^3+^ for subsequent adsorption experiments, adsorption experiments were carried out using 0.1 M hydrochloric acid Gd^3+^ solutions with an adjusted pH of 2.0–7.0 with three different aerogels of CNCs, c-CNCs, and c-CNCs@N, and the experimental setting was 10 mL of Gd^3+^ stock solution with an initial concentration of 50 mg L^−1^, adsorption time of 24 h, and adsorbent content of 10 mg. Considering the transformation of Gd^3+^ into Gd(OH)_3_ precipitate under alkaline conditions, the pH range of 2.0–7.0 was chosen for the test. The test results are shown in Figure 5. It can be seen that the adsorption effect of the three aerogels, pure CNCs, c-CNCs, and c-CNCs@N, on gadolinium ions increased, while the adsorption performance of the c-CNCs and c-CNCs@N aerogels improved compared with that of the CNCs aerogels, which indicates that the good pore structure is still preserved after carbonization to improve the adsorption performance. Because the adsorption capacity of the pure CNCs is relatively low, we did not consider subsequent experiments, in which we would only compare the adsorption capacities of the c-CNCs and c-CNCs@N. With the increasing pH, the adsorption capacity of the material also increased, and we measured the selective adsorption capacity of the aerogels according to the ratio of the adsorption capacity of the c-CNCs and c-CNCs@N, and the maximum value was 1.12 at pH = 5.0. It is also obvious from the figure that the adsorption capacity increased slowly at a pH less than 5.0, increased substantially when the pH was equal to 5.0, and increased faster when the pH was greater than 5.0. This is probably due to the decrease in carboxymethyl dissociation, which leads to a decrease in the adsorption performance of the aerogels for gadolinium ions, so we chose pH 5.0 for the subsequent adsorption experiments.

#### 2.2.2. Adsorption Kinetics Experiment

Adsorption kinetic tests were performed on the adsorption materials to investigate when the aerogel materials reached adsorption equilibrium during the adsorption process. The experimental environment was at 298 K, pH = 5.0, and an initial Gd^3+^ concentration of 50 mg L^−1^, and 13 experimental data were selected for sampling and ICP testing to obtain the remaining Gd^3+^ concentration within 0–1440 min. A quasi-first-order kinetic model (PFOKM) and a quasi-second-order kinetic model (PSOKM) were used to perform the nonlinear fitting of the kinetic experimental data.

**Figure 6:**
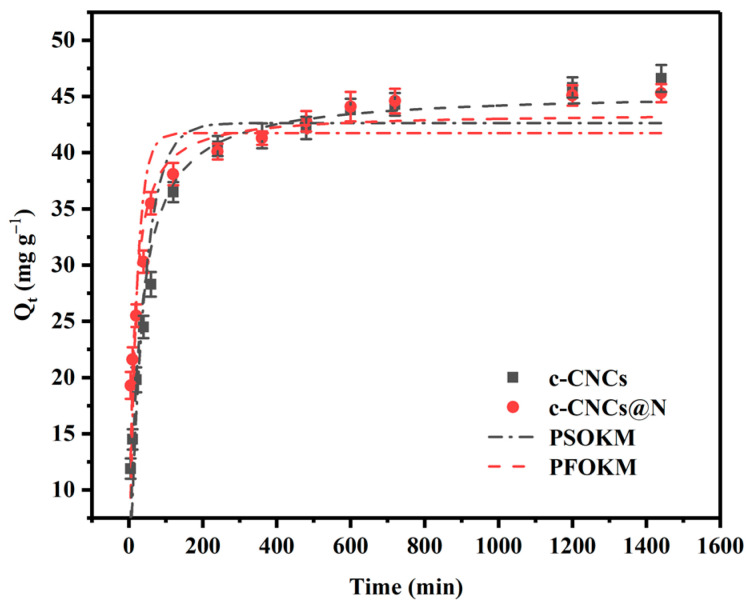
Kinetics of c-CNCs and c-CNCs@N aerogel adsorbing Gd^3+^.

As shown in Figure 6, we can clearly see from the figure that both the c-CNCs and c-CNCs@N aerogels increased the adsorption effect on Gd^3+^ rapidly within 0–60 min. c-CNC adsorption reached the equilibrium at about 800 min, while the c-CNCs@N adsorption equilibrium occurred 300 min earlier, and the adsorption amount of c-CNCs@N aerogel is larger than that of the c-CNCs from 5 min, and the rise in the adsorption rate occurred faster. This indicates that the nitrogen doping process not only increases the selectivity of the material, but also relatively accelerates the adsorption rate of the material. The relevant kinetic adsorption fitted data are shown in Table 1, and it can be seen that the fitted data with correlation coefficients R^2^ greater than 0.99 is a quasi-second-order kinetic model, which indicates that the adsorption process of the aerogel material is mainly based on chemisorption, supplemented by physical adsorption.

#### 2.2.3. Adsorption Isotherm Experiment

**Figure 7:**
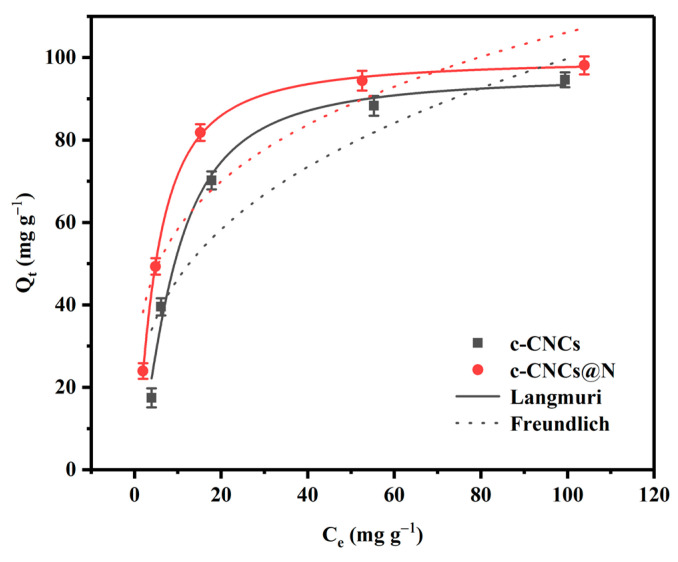
Adsorption isotherm c-CNCs and c-CNCs@N.

In order to obtain the maximum adsorption capacity of the materials, the materials were exposed to different concentrations of Gd^3+^, and the adsorption differences as well as the maximum adsorption capacity of the different materials were analyzed. The specific data obtained were fitted to the data using the two common isothermal models of Langmuir and Freundlich, and the results of the fitting are shown in Figure 7, where the adsorption capacity of the materials increased with the increase in the initial concentration of Gd^3+^. The maximum adsorption capacities of the c-CNCs and c-CNCs@N aerogel materials were 95.63 and 99.65 mg g^−1^, respectively. The adsorption capacities of the c-CNCs@N aerogel were consistently higher than those of the c-CNCs aerogel, which indicates that the adsorption capacities of the materials also increased after doping. The relevant isotherm constants are summarized in Table 2. The R^2^ values greater than 0.99 indicate that both the materials conform to the Langmuir model, which represents that the adsorbent materials are monolayers in the adsorption process. c-CNCs@N has a lower 1/n value, which represents that the material has a better adsorption capacity for Gd^3+^ and has better adsorption conditions.

#### 2.2.4. Adsorption Thermodynamics Experiment

**Figure 8:**
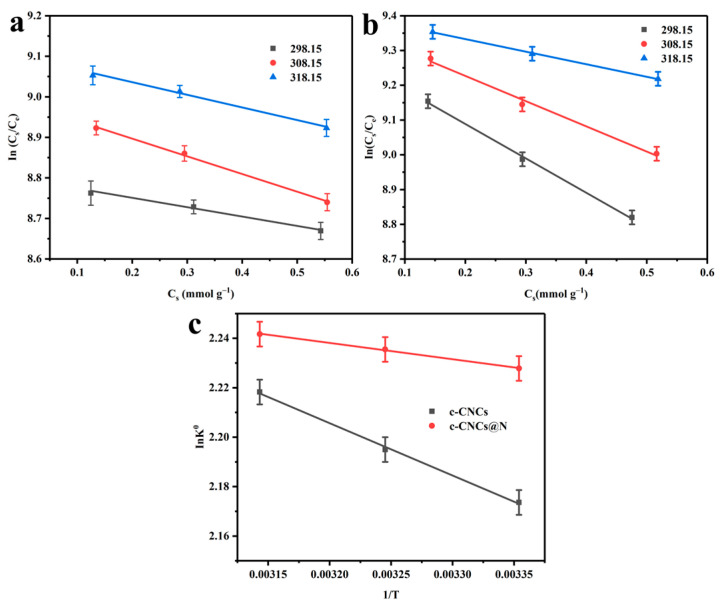
Thermodynamic fitting curve of c-CNCs and c-CNCs@N aerogel adsorbing Gd^3+^. Thermodynamic fitting curve for adsorption of Gd^3+^ for (**a**) c-CNCs, (**b**) c-CNCs@N: ln(Cs/Ce) diagram, (**c**) Thermodynamic fitting curves for adsorption of Gd^3+^ by c-CNCs, c-CNCs@N: lnK° and 1/T.

We selected three temperatures of 298.15 K, 308.15 K, and 318.15 K for the adsorption experiments on the c-CNCs and c-CNCs@N aerogel materials, respectively, and the experimental data were linearly fitted, as shown in Figure 8, where a, b represents the effect of both aerogels on adsorption at different temperatures, and ∆G° was calculated according to the Gibbs free energy equation. Panel c shows the relationship between lnK° and 1/T to obtain ΔH° and ΔS°, and the thermodynamic parameters obtained are shown in Table 3, from which it can be seen that ∆G° is negative, which indicates that the adsorption process of aerogel materials is a spontaneous process. The fact that ∆H is greater than 0 indicates that the adsorption process of the aerogel material is heat-absorbing, which suggests that the rate of the reaction is enhanced with an increasing temperature. This is also consistent with the experimental data obtained. In conclusion, the adsorption of Gd^3+^ by the c-CNCs and c-CNCs@N aerogel materials is a spontaneous and heat-absorbing process.

#### 2.2.5. Repeatable Adsorption Experiment

**Figure 9:**
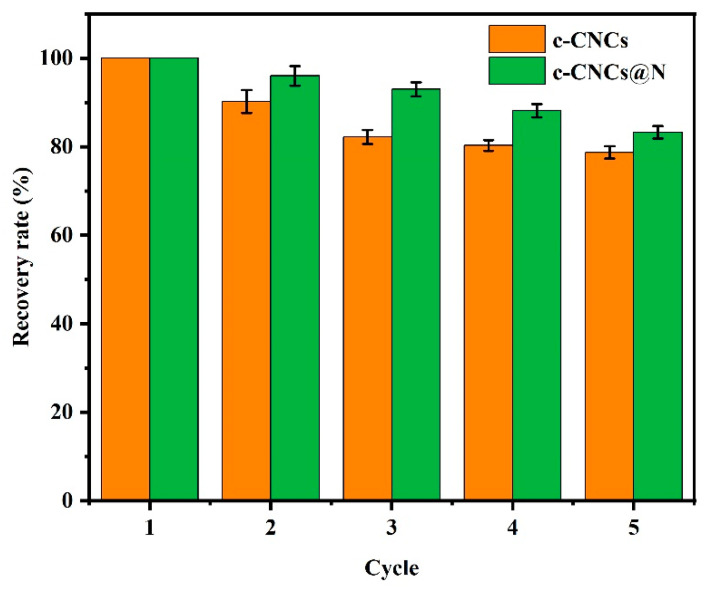
Repeated adsorption experiment of c-CNCs and c-CNCs@N on Gd^3+^.

To investigate the practical application of the aerogel materials, five reuse cycles were conducted for the c-CNCs and c-CNCs@N aerogel materials, and the experiment was eluted with 9:1 acetic acid mixture after the adsorption of Gd^3+^. The experiment was repeated under the same conditions, and in this way, the experiments were repeated five times, and the adsorption data obtained are shown in Figure 9. It can be seen from the figure that the adsorption performance of both the c-CNCs and c-CNCs@N aerogels decreased after five cycles, mainly because some Gd^3+^ remained on the adsorption sites as well as in the pore channels during the elution process, and acid elution may also lead to the loss of carboxyl and carboxymethyl groups by shedding. However, the c-CNCs and c-CNCs@N aerogels still maintained 78.71% and 83.27% of the original adsorption capacity after five cycles, respectively. The aerogel materials have good reusability, which will greatly reduce the cost of practical applications.

#### 2.2.6. Selective Adsorption Experiment

**Figure 10:**
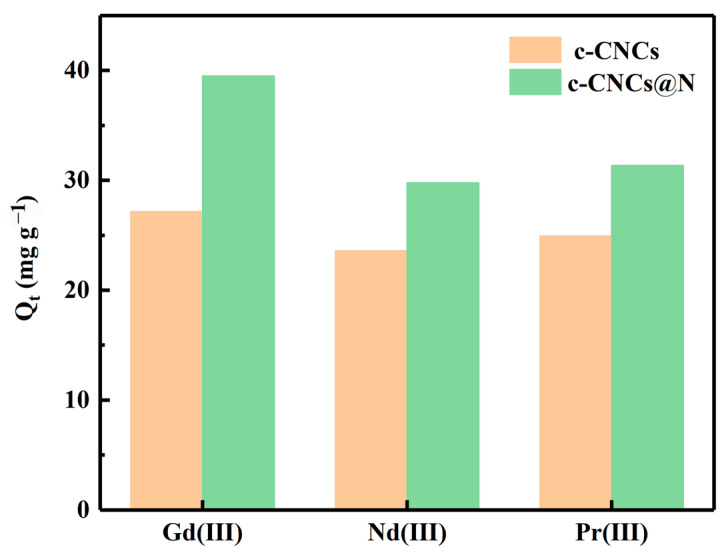
The adsorption amount of different rare-earth ions adsorbed by adsorbents.

We selected two rare-earth ions with similar physicochemical properties to gadolinium for the adsorption selectivity experiments, praseodymium (Pr) and neodymium (Nd), respectively. We placed 10 mg of the c-CNCs and c-CNCs@N aerogel materials into solutions containing Nd^3+^, Pr^3+^, and Gd^3+^ prepared from Gd(NO_3_)_3_·6H_2_O, Nd(NO_3_)_3_·6H_2_O, and Pr(NO_3_)_3_·6H_2_O, each with the same three ionic concentrations and a pH of 5, and reacted them for 24 h; the ion concentrations in the remaining solutions after the reaction were detected using ICP. The corresponding K_d_ values were calculated according to equation. The K_d_ values reflect the adsorption selectivity of the adsorbent for Gd^3+^. The results are shown in Table 4 and integrate the data shown in Figure 10. It is obvious from the figure that the c-CNCs have a certain adsorption effect on the ions other than the target ion with similar ion interference and poor selective adsorption ability of the target ion. And the maximum value of Kd for c-CNCs@N reached 3757.37 mL g^−1^. This suggests that the nitrogen-doped carbon aerogels (c-CNCs@N) exhibit a certain selectivity for Gd^3+^, which greatly reduces the difficulty of recovering Gd^3+^ in industries and provides a feasible solution for the recovery of rare-earth elements.

## 3. Experimental and Methods

### 3.1. Materials

Skimmed cotton was purchased from a pharmacy. Ammonia (NH_4_OH, 25%), urea, ethanol (C_2_H_5_OH, 98%), monochloroacetic acid (ClCH₂COOH), isopropanol (C_3_H_8_O), methanol (CH_3_OH), sulfuric acid (H_2_SO_4_), sodium hydroxide (NaOH), and gadolinium nitrate (Gd(NO_3_)_3_·6H_2_O) were purchased from Shanghai Aladdin Biochemical Technology Co. All the reagents were configured with distilled water (0.1 × 10^6^ Ω·cm).

### 3.2. Preparation of Cellulose Nanocrystal (CNCs)

Bar CNCs were obtained based on our previous study [25,26]. Briefly, concentrated sulfuric acid (75 mL) was slowly added to water (75 mL), stirred well, and cooled to room temperature. A total of 10 g of skimmed cotton was put into the mixture and stirred for 3 h at 45 °C in an oil bath. It was poured into ice water (1500 mL) and left to settle for 12 h. The upper clear part was poured out, and the lower suspension was centrifuged 5–10 times at 10,000 rpm and purified by dialysis to a pH value greater than 2.4 to obtain a white suspension. The final concentration of the white suspension was adjusted to 3.0 wt% in the spin flask and was labeled as CNCs.

### 3.3. Preparation of Carboxymethylated Cellulose Nanocrystals (c-CNCs)

The CNCs carboxymethylation method was based on that reported in the literature with minor modifications [27,28]. The CNCs were taken and centrifuged at 10,000 rpm for 10 min, the supernatant was poured out, ethanol was added to the centrifuge tube set to the 10 mL mark and stirred well, and the centrifugation was cycled 4 times after 20 min of resting, and ethanol was used as a solvent instead of solvent in the CNCs. A total of 11 g of CNCs solution with 50 mL of ethanol solvent was impregnated in a mixture of 1 g of monochloroacetic acid and 50 mL of isopropanol for 30 min. Then, in a reaction vessel equipped with a condenser tube, it was added to a solution of 50 mL of methanol and 200 mL of isopropanol with 1.62 mg of NaOH and heated to 55 °C for 1 h to finally obtain the carboxymethylated CNCs (c-CNCs).

### 3.4. Preparation of Nitrogen-Doped Carboxymethyl Cellulose Nanocrystals (c-CNCs@N)

**Scheme 1:**
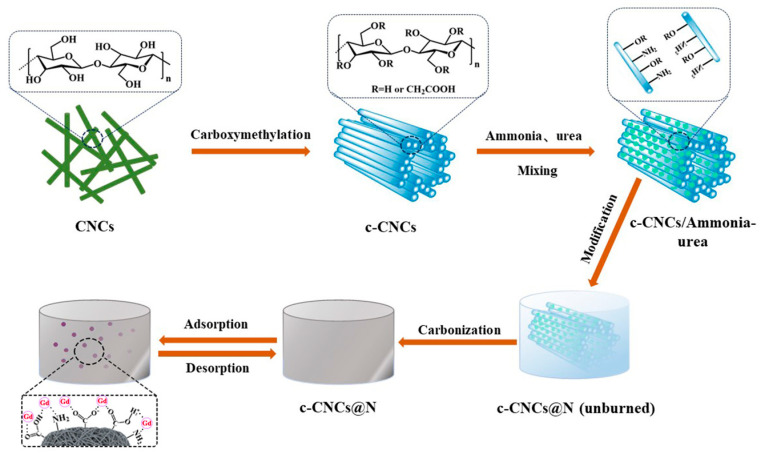
Flowchart of the preparation of c-CNCs@N aerogel using the mechanism of gadolinium ion adsorption.

The aqueous slurry formed from carboxymethyl cellulose was dispersed in an aqueous ammonia–urea solution (5 mL of ammonia and 4 g of urea dissolved in 5 mL of deionized water) and sonicated for 30 min. The mixture was placed in a certain amount of ethanol for one week to displace the water in the mixture and form a wet gel, and the wet gel was supercritically dried by CO_2_ to obtain the cellulose aerogels. The aerogels were purged in a tube furnace using nitrogen at a rate of 150 mL·min^−1^ for 15 min, heated at 150 °C for 30 min to remove water and volatiles, and the decomposition of cellulose and lignin was carried out at 450 °C for 30 min to obtain nitrogen-doped carbon cellulose aerogels (c-CNCs@N). The preparation process and possible adsorption mechanism of the aerogels are shown in Scheme 1.

### 3.5. Adsorption Experiment

A series of static adsorption experiments, including the determination of the optimal adsorption pH, adsorption kinetics, adsorption thermodynamics, adsorption isotherms, and reproducible adsorption experiments, were performed on the successfully synthesized adsorbent materials as well as the comparison materials. The final experimental results were measured by inductively coupled plasma photoemission spectrometry (ICP), and graphs were calculated and fitted to the results using relevant software (1991–2016 OriginLab Corporation, Northampton, MA, USA) for analysis and discussion.

### 3.6. Characterization Instruments

The morphology and microstructure of the c-CNCs@N materials were observed by scanning electron microscopy (SEM, Hitachi, S-4800II, Hitachi Group, Tokyo, Japan). The functional groups in the molecules of c-CNCs@N materials were observed in the range of 4000–500 cm^−^^1^ using a Fourier-transform infrared spectrophotometer (FTIR, Nicolet NEXUS-470 FT-IR apparatus, Thermo Fisher Scientific, Waltham, MA, USA). The specific surface area of c-CNCs@N materials was determined by the N_2_ adsorption–desorption method using an automatic specific surface area and porosity analyzer (Micromeritics Instrument Corporation, Atlanta, Georgia). The surface charge of c-CNCs@N materials was determined using a zeta potential meter (Malvern, Malvern, UK). The materials were analyzed with the above characterization instruments to ensure the successful synthesis of the materials.

## 4. Conclusions

In this study, we used the ammonia–urea system to induce a carboxymethyl cellulose aerogel to be able to form defects by stable exfoliation during the carbonization process. The c-CNCs@N aerogel with an excellent performance and large specific surface area was successfully prepared with supercritical CO_2_ drying technology, achieving the efficient and selective adsorption separation of Gd^3+^. The successful synthesis, as well as the physicochemical properties of the materials, were demonstrated by relevant characterization. The results of static adsorption experiments showed that the maximum adsorption capacity of the c-CNCs@N was 99.65 mg g^−1^ at pH = 5.0 and had good reusability. The kinetic and isotherm fitting curves indicated that the adsorption process of the aerogels was dominated by monolayer chemisorption and supplemented by physical adsorption. In conclusion, the method in this study can be a feasible way for the industrial recovery of Gd^3+^.

## Figures and Tables

**Table 1:** PFOKM and PSOKM parameters of c-CNCs@N and c-CNCs@N.

Absorbents	Q_e,exp_ (mg g^−1^)	Quasi-First-Order Kinetic Model			Quasi-Second-Order Kinetic Model				
		Q_e,c_ (mg g^−1^)	K_1_ (min^−1^)	R^2^	Q_e,c_ (mg g^−1^)	K_2_ × 10^−3^(g mg^−1^ min^−1^)	h (mg g^−1^ min^−1^)	t_1/2_ (min)	R^2^
c-CNCs	46.6	42.64	0.0248	0.904	45.36	0.083	1.709	26.53	0.991
c-CNCs@N	45.3	41.75	0.05047	0.763	43.53	0.192	3.636	11.97	0.992

**Table 2:** Langmuir and Freundlich parameters of c-CNCs and c-CNCs@N.

Absorbents	Langmuir Isotherm Equation			Freundlich Isotherm Equation		
	Q_m_ (mg g^−1^)	K_L_ (L mg^−1^)	R^2^	K_F_ (mg g^−1^)	1/n	R^2^
c-CNCs	95.63	0.0373	0.996	21.455	0.334	0.848
c-CNCs@N	99.65	0.12967	0.998	32.134	0.259	0.798

**Table 3:** Gd^3+^-adsorption thermodynamic parameters.

Absorbents	Δ*H*° (kJ mol^−1^)	Δ*S*° (J mol^−1^)	T (K)	K°	Δ*G*° (kJ mol^−1^)	R^2^
c-CNCs	211.69	2.88	298.15	8.79	−5.39	0.998
			308.15	8.98	−5.63	
			318.15	9.10	−5.84	
c-CNCs@N	65.98	2.45	298.15	9.28	−5.52	0.998
			308.15	9.37	−5.73	
			318.15	9.41	−5.93	

**Table 4:** The *K*_d_ value of Gd^3+^ adsorbed by aerogels in the mixed solution.

Cation	c-CNCs		c-CNCs@N	
	*C*_f_ (mg L^−1^)	*K*_d_ (mL g^−1^)	*C*_f_ (mg L^−1^)	*K*_d_ (mL g^−1^)
Gd^3+^	22.86	1187.23	10.51	3757.37
Nd^3+^	26.41	893.22	20.23	1471.58
Pr^3+^	25.07	994.32	19.64	1545.82

## Data Availability

Data are contained within the article.
